# A Safety-II approach to learn from practice variation in the diagnostic process in the emergency department: an action research study

**DOI:** 10.1515/dx-2025-0087

**Published:** 2025-10-13

**Authors:** Rick Roos, Laura Zwaan, Myrthe Maranus, Iwan A. Meynaar, Vanessa J. Valk, Cees van Nieuwkoop, Gert-Jan Kamps, Hardeep Singh, Maarten O. van Aken

**Affiliations:** Department of Internal Medicine, Haga Teaching Hospital, The Hague, The Netherlands; Health Campus The Hague/Department of Public Health and Primary Care, Leiden University Medical Center, The Hague, The Netherlands; Institute of Medical Education Research Rotterdam, Erasmus Medical Center, Rotterdam, The Netherlands; Department of Intensive Care, Haga Teaching Hospital, The Hague, The Netherlands; Department of Emergency Medicine, Haga Teaching Hospital, The Hague, The Netherlands; Intergo, Human Factors and Ergonomics, Utrecht, The Netherlands; Center for Innovations in Quality, Effectiveness and Safety, Michael E. DeBakey Veterans Affairs Medical Center, Houston, TX, USA; Health Services Research, Baylor College of Medicine, Houston, TX, USA

**Keywords:** Safety-II approach, diagnostic process, practice variation, emergency department, action research, methodology

## Abstract

**Objectives:**

The focus on improving patient safety has mainly been by learning from errors and near misses (Safety-I). We applied a novel Safety-II approach to identify and learn from practice variations in the diagnostic process in the emergency department (ED), and subsequently design and implement practice changes.

**Methods:**

In this single-center study, we used action research (cycles of micro-experiments to study and improve processes with active stakeholder involvement) in the diagnostic process. We used three subsequent observation cycles of the following six steps: observations, gathering of follow-up data, analyses, a co-creation session with involved stakeholders, sharing of best practices, and updating the observation form. The observations and analyses focused on identifying practice variation in everyday practice rather than on what went right or wrong. During co-creation sessions, stakeholders discussed whether practice variations were reflective of possible improvements in the diagnostic process. Promising best practices were identified, and subsequently implemented as practice changes aiming to improve the diagnostic process. Implemented practice changes were evaluated in subsequent cycles.

**Results:**

Forty diagnostic processes were observed. We identified practice variations that reflected the resilience and adaptability of clinicians, as well as variations revealing opportunities to improve the diagnostic process. Five identified best practices were implemented as practice changes: a template for the documentation of ED preannouncements, a document with relevant digital information resources for residents, face-to-face supervision by Internal Medicine consultants during office hours, blood sampling at triage, and adding lipase to the standard ED blood tests. These changes were well-received by stakeholders, also shown by an adoption-rate of 67–100 % of observed cases after implementation.

**Conclusions:**

A Safety-II approach with action research and direct observations of the diagnostic process in the ED can be successfully applied to identify and learn from practice variation, and can lead to well-received practice changes.

## Introduction

Most people will experience a diagnostic error in their lifetime [1]. Studies indicate diagnostic error rates ranging from 5 to 15 % [2], [3], [4]. Diagnostic errors are often considered preventable, and their consequences are serious as they more often result in readmissions, morbidity and mortality [5], [6], [7]. Factors contributing to diagnostic errors are often related to the follow-up of abnormal test results or the failure to consider the correct diagnosis [8], 9]. Studies have shown that diagnostic discrepancies often occur at the emergency department (ED), but are also reflective of the complex nature of the diagnostic process and the ED [10], 11]. The study of diagnostic errors in clinical practice remains a methodologic challenge [12].

Current approaches to study diagnostic errors mostly involve retrospective analyses and learning mainly occurs from what goes wrong (e.g. adverse events and near misses) [13], 14]. This approach, known as the Safety-I approach, is based on the predictability of processes and presumes things go wrong because of identifiable failures [15]. Retrospective evaluation of diagnostic errors is difficult and susceptible to hindsight bias [16]. The diagnostic process is a dynamic, team-based activity that involves uncertainty, evolves over time, and requires effective communication and collaboration among multiple providers, diagnostic services, and the patient [17]. This makes it one of the most complex and unpredictable processes in medicine. In most cases, it is difficult to establish at what moment in time the disease could and should have been diagnosed [18]. Therefore, how the diagnostic process takes place (work-as-done) often deviates from standard operating procedures and guidelines (work-as-imagined).

The Safety-II approach assumes that variations in everyday practice reflect adaptations needed to respond to varying conditions, especially in complex processes. In contrast to the Safety-I approach, this variability (resilience) is considered the reason why things go right [15]. The aim of this approach is to improve everyday work by learning from best practices while allowing for variability in the process, thereby taking into account the complexity of (healthcare) processes that require continuous adaptations from the (medical) personnel. Therefore, the Safety-II approach could be valuable in the complex and adaptive context (e.g. a busy ED) of the diagnostic process. Furthermore, Safety-II not only enables learning from what went wrong, but also learning from what went well, thereby expanding the number of cases to learn from. Safety-II can thus complement Safety-I. However, few practical approaches exist to operationalise Safety-II and more research on its effectiveness is needed [19], 20]. These approaches include: observations of everyday work, action research, and the Functional Resonance Analysis Method (FRAM) [21].

Action research distinguishes itself from other forms of research by its dynamic process of changing and producing knowledge that takes place in the present tense and where data emerges through intervention and reflection-in-action, and it aims at contributing both practice and theory [22]. During this process, stakeholders are empowered to get engaged with research and subsequent development and/or implementation of interventions. An example is an action research study using video-reflexive ethnography that led to a reshape of the Intensive Care Unit ward round practices [23]. Action research matches well with the Safety-II principles due to its explorative design, and contribution to a learning culture.

In this action research study, we explored the use of the Safety-II approach in the diagnostic process in the ED by directly observing the diagnostic process to identify everyday practice variations, and subsequently design and implement practice changes based on identified best practices.

## Materials and methods

### Design

A single-center action research study with micro-experiments to identify best practices in the diagnostic process followed by the implementation and evaluation of practice changes [24]. This approach differs from traditional quality improvement methods (e.g. Plan-Do-Act-Study cycles) as these approaches start with predefined problems, while this approach is inductive as we started observing daily practice with the aim to identify practice variation.

The study consisted of a preparation phase followed by three subsequent observation cycles during which the diagnostic process of residents at the ED was directly observed to identify practice variation [25], 26]. This practice variation was discussed in co-creation sessions with stakeholders leading to the identification of best practices. Based on these identified best practices, practice changes were designed and implemented to improve the diagnostic process, and were evaluated in subsequent observation cycle(s).

### Setting

The study was carried out at one of the largest EDs in the Netherlands, with approximately 45.000 presentations annually, from November 2021 until July 2022.

In the Netherlands, patients are often referred to the ED by general practitioners (GPs) as they act as gatekeepers to the wider Dutch healthcare system. GPs frequently make a preannouncement phone call to the consultant as part of the referral of a patient to the ED. Based on their medical urgency, patients arrive at the ED either by own transportation or an ambulance. At the ED, patients arriving by ambulance receive an ED room immediately. Patients arriving with their own transportation are placed in the waiting room, triaged according to their medical urgency, and moved to an ED room based on their medical urgency and room availability. At the ED, patients are primarily evaluated by nurses and residents, the latter subsequently discussing the case with their supervising consultant(s).

### Participants

There were three types of participants: patients, residents, and stakeholders.

#### Patients

Patients presenting to the ED were eligible for participation when they met the following inclusion criteria: age ≥18 years and primarily presenting to the ED for the Internal Medicine or Emergency Medicine specialties, with complaints of abdominal pain, chest pain, dyspnea and/or fever. These clinical presentations were chosen because of their broad differential diagnosis, and therefore increase the likelihood to observe practice variation in the diagnostic process.

On observation days, the first eligible patient presenting to the ED was asked for written informed consent to be observed during their ED stay and the permission to access to their electronic health record (EHR), upon his/her arrival at the ED. In case the first patient did not want to participate, the second eligible patient was asked for informed consent. All patients that provided written informed consent were subsequently observed, and when the observation was finished, the next eligible patient was asked for informed consent.

#### Residents

Residents were eligible to participate if their speciality was Internal Medicine or Emergency Medicine. Residents were informed about the study during the preparation phase, and were asked for their written informed consent to be observed at the start of their shift.

#### Stakeholders

A diverse group of 25 stakeholders participated in the study during co-creation sessions to function as a sounding board and critical partner. During every co-creation session, 12–16 stakeholders were present. This group consisted of individuals with different roles in the diagnostic process: consultants and residents (Internal Medicine and Emergency Medicine), ED nurses, patient-representatives, members of the Quality and Safety board, and the study supervisors (RR, LZ, MM, IM, VV, CvN, GK and MvA). All consultants, residents and ED nurses were part of the staff working at the ED, and thereby experienced the impact of the changes made in each observation cycle on a daily basis.

### Materials

A standardised observation form was developed iteratively to objectively document information associated with the diagnostic process (see Supplemental Appendix 1). A first draft was developed by the study supervisors based on a model of the diagnostic process [1]. This version was revised based on the input of stakeholders in the kick-off co-creation session and two pilot observations, and subsequently used in the first observation cycle.

The observation form included an ‘other’ section for observers to report additional factors or circumstances that potentially influenced the diagnostic process. Every observed practice that potentially influenced the diagnostic process was considered a relevant finding. On the observation form, the exact time of all actions (e.g. supervision) was documented, thereby enabling the analysis of work-as-done using a chronological storyline. As contextual factors are known to influence the diagnostic process, the context in which actions took place in the diagnostic process was also documented on the form (e.g. face-to-face or by phone) [27].

### Procedures

**Figure 1:**
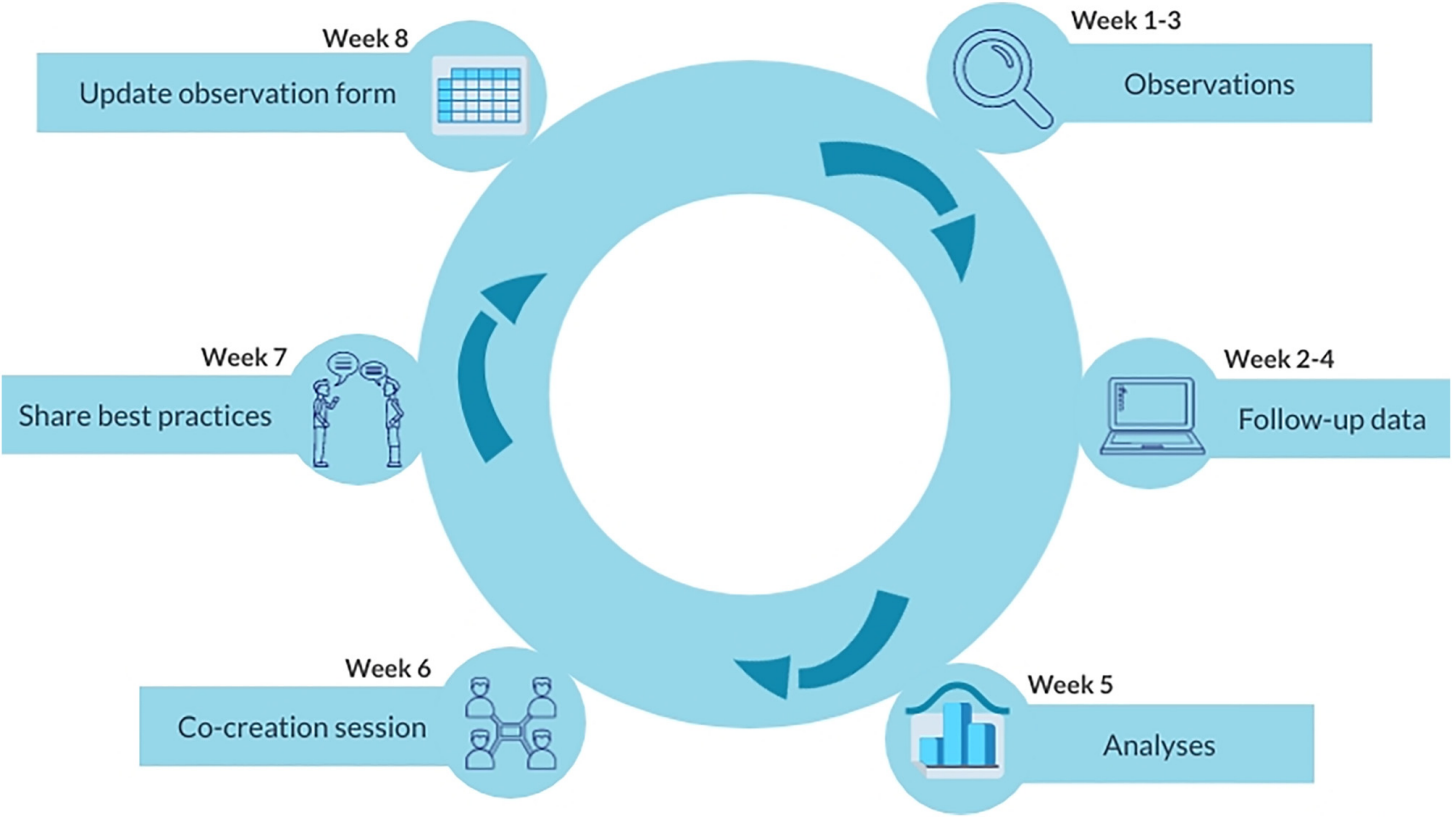
The six steps of each observation cycle.

The study consisted of a preparation phase and three subsequent observation cycles. Each observation cycle consisted of the same six steps (see Figure 1). The preparation phase took two months in total. Each observation cycle took 8 weeks (two months).

#### Preparation phase

At the start of this project, a kick-off co-creation session was held with the stakeholder group to discuss the project aims and the observation form. We aimed to receive input, support and commitment to the project [28], 29]. After this session, two pilot observations were performed before finalising the observation form.

**Step 1:**

**Observations**

During the first 3 weeks of an observation cycle, the aim was to observe the diagnostic process in 15 patients. Observations took place at the ED during day and evening shifts. The observers (medical student MM and Internal Medicine resident RR) followed residents from the arrival of the patient at the ED until ED disposition (hospitalisation or discharge), and collected quantitative and qualitative data using the observation form.

**Step 2:**

**Follow-up data**

One week after every observed diagnostic process (week 2–4 of each observation cycle), the EHRs of observed patients were reviewed to collect follow-up data. In patients discharged home from the ED, these data consisted of whether the patient revisited the ED, and the definitive results of additional diagnostics (e.g. blood cultures). If a discharged patient did not revisit the ED, no additional follow-up data were collected, and it is therefore unknown whether the working diagnosis at the ED was correct or not. In hospitalised patients and discharged patients revisiting the ED, follow-up data was collected on the working diagnosis 1 week later, thereby enabling comparison with the initial working diagnosis at the ED.

**Step 3:**

**Analyses**

In the fifth week of each cycle, all gathered data were described in a chronological storyline from ED arrival until ED disposition. Each storyline represented the observed work-as-done of the diagnostic process. Storyline comparison revealed practice variations between the observed diagnostic processes (e.g. in method and frequency of supervision of residents). Subsequently, identified practice variations were discussed by the study supervisors, and the most relevant practice variations were selected for the co-creation sessions based on the experiences of the study supervisors, and the expected clinical impact on the diagnostic process (e.g. logistical process, availability of clinical information and test results, and communication).

**Step 4:**

**Co-creation session**

The most relevant practice variations were discussed with stakeholders in the co-creation session in week six of each cycle. The co-creation sessions took place in a meeting room near the ED, facilitating participation of the ED staff. Stakeholders could attend in person or online, though 90–95 % joined in person. Each session was scheduled for 1.5  hours and followed a predefined format, starting with a brief overview of the project aims and design, followed by an evaluation of previously implemented changes, and a presentation of the newly observed practice variations. Most time was dedicated to a moderated discussion on the newly observed practice variations. The moderator (MM or RR) stressed the criteria of relevance and impact, but the perspective of the stakeholder group was leading in the final selection as their support for the improvements was considered crucial for the adoption in daily practice. The moderator encouraged all stakeholders to share their insights on which variations could contribute to the improvement of the diagnostic process. After reaching consensus on best practices and their feasibility, stakeholders collaboratively designed the practice changes for integration into the clinical workflow. For the informal small-scale evaluation of implemented practice changes from previous cycles, the observations and the experiences (practical use and satisfaction) of involved stakeholders (ED staff) were discussed. Based on these discussions, practice changes were adapted where necessary.

**Step 5:**

**Share best practices**

In the seventh week of every observation cycle, the co-designed practice changes were shared with all involved personnel through short oral presentations, newsletters, and e-mails. The stakeholders were actively involved in sharing best practices with the aim of promoting the uptake of the practice changes into everyday work among their colleagues.

**Step 6:**

**Update observation form**

In all observation cycles, the observation form was updated in the eight week. Implemented practice changes were added to the form to enable evaluation in subsequent cycles.

## Results

In three observation cycles, the diagnostic process was observed in 40 patients (50 % female, median age 67 years, see Table 1), 10 patients in cycle 1, and 15 patients in cycles 2 and 3. In cycle 1, the number of observations was limited to 10 due to a longer observation time per case than expected. In these 40 cases, 25 different residents (15 Internal Medicine and 10 Emergency Medicine) were observed. Abdominal pain was the most common reason (40 %) for ED presentation. Nineteen patients were hospitalised, and one patient revisited the ED after initial discharge. Of these 20 patients (50 %) with follow-up data, the working diagnosis changed in four patients during hospitalisation, and in the patient revisiting the ED.

**Table 1:** Characteristics of the patients in the observed diagnostic processes.

	Patients (n=40)
Age, median [IQR]	67 [51–83]
Female, % (n)	50 % (n=20)
Presenting complaint, % (n) Abdominal pain Chest pain Dyspnoea Fever	40 % (n=16) 2.5 % (n=1) 25 % (n=10) 32.5 % (n=13)
Specialty resident, % (n) Emergency Medicine Internal medicine	25 % (n=10) 75 % (n=30)
ED disposition, % (n) Discharge Hospitalisation	52.5 % (n=21) 47.5 % (n=19)
Correct working diagnosis on ED, % (n)^a^ Correct Incorrect Unknown	37.5 % (n=15) 12.5 % (n=5) 50.0 % (n=20)

^a^No follow-up data was available in 20 patients after initial discharge from the emergency department, and it is therefore unknown whether the working diagnosis was correct. ED, emergency department; IQR, interquartile range.

### Observation cycle 1

The first observation cycle revealed practice variation in how the preannouncement of the referral was handled. In one out of 10 patients, information on the preannouncement was documented in the EHR, which included presenting complaints, the referring physician, the medical history and suggested additional diagnostics. In the other nine cases, the information of the preannouncement was transferred by a phone call to the resident, with a limited amount of the aforementioned information (see Table 2). During the co-creation session, stakeholders identified the documentation of information about the preannouncement in the EHR as a best-practice, as it could prevent the loss of data and save time in the diagnostic process. To apply this in clinical practice, a standardised EHR template for the preannouncement was identified as a feasible practice change. The stakeholders identified seven items to be documented in this template, named the ‘Successful Seven’ (see Supplemental Appendix 2). This template was embedded in the EHR and shared with all involved personnel.

**Table 2:** The five practice changes developed using the observed practice variation in the diagnostic process, the input from the stakeholders during the co-creation session, and the identified best-practice based on the co-creation session.

Cycle	Theme of variation	Observed practice variation	Input co-creation session	Identified best practice	Practice change
1	Communication of a referral	– No documentation or phone call from the consultant about the referral – Phone call from consultant with varying information about the referral – Documentation in EHR and phone call by consultant with information about referral	– Residents often miss information, where consultants are unaware of – Documentation costs time but could also save time in diagnostic process – Documentation could prevent loss of data (e.g. handovers between shifts)	Documentation of information about the preannouncement in the EHR	Template for documentation of preannouncements in the EHR (successful seven)
1	Use of information resources	– Residents used digital information resources in 7/10 cases (4 used multiple) – A variety of digital information resources was used (e.g. UpToDate)	– The used information resources were often not supported by consultants – The correct hospital protocols are often difficult to find	Use of resources/guidelines supported by consultants	Document with relevant digital information resources for residents
2	Supervision method/timing	– Emergency Medicine consultants supervise face-to-face and often before results are in – Internal Medicine residents often call their consultant when all results are in	– Easier to ask for supervision before results are in with consultant in ED – Supervision earlier in the process could speed up next steps in process	Physical presence of Emergency Medicine consultants in the ED	Face-to-face supervision by Internal Medicine consultants during office hours
2	Timing of taking of blood samples	– Patients waited for hours after triage without blood samples taken – Fastest two lead times were when blood samples were taken in triage (2/7 patients)	– Difficult to obtain blood samples in triage when the waiting room is busy – Obtaining blood samples in triage could save time later in the process	Taking of samples as early in the process as possible (e.g. triage)	Obtaining blood samples in triage
3	Timing of lipase testing	– Triagist added lipase to laboratory package in one patient with abdominal pain – Lipase was tested later in the process in four patients with abdominal pain	– Testing lipase in patients with upper abdominal pain is standard practice – Adding lipase to standard laboratory package could save time on the ED	Testing lipase in patients with abdominal pain at the ED	Addition of lipase to the standard blood tests for all patients at the ED

ED, emergency department; EHR, electronic health record.

Furthermore, practice variation was seen in the use of digital information resources with some residents not using digital information resources related to the diagnosis or treatment while others used resources varying from international subscription databases (UpToDate) to national/local guidelines, external guidelines and Google searches. During the co-creation session, stakeholders reached consensus that a document with relevant digital information resources, supported by consultants, could facilitate the diagnostic process. A document with relevant digital information resources was created through input from six consultants from different subspecialties and subsequently shared with all involved personnel.

### Observation cycle 2

Practice variation was observed in the extensiveness of the information documented in the EHR template with some consultants e.g. documenting intended diagnostics. Discussing this practice variation, residents and nurses stated that the diagnostic process went particularly smooth when intended diagnostics were documented in the ‘Successful Seven’ as they were more aware what additional samples needed to be taken upon ED arrival of the patient. Consensus was reached to imbed intended diagnostics in the ‘Successful Seven’ template in the EHR. All involved personnel was informed about the change in the EHR template.

Besides that, practice variation was seen in the method and frequency/timing of supervision of residents (see Table 2). In the co-creation session, the physical presence of Emergency Medicine consultants on the ED was identified as a best-practice, and consensus was reached that face-to-face supervision by Internal Medicine consultants was feasible and can be beneficial to the diagnostic process. All Internal Medicine consultants and residents were informed about this practice change in ED supervision.

**Figure 2:**
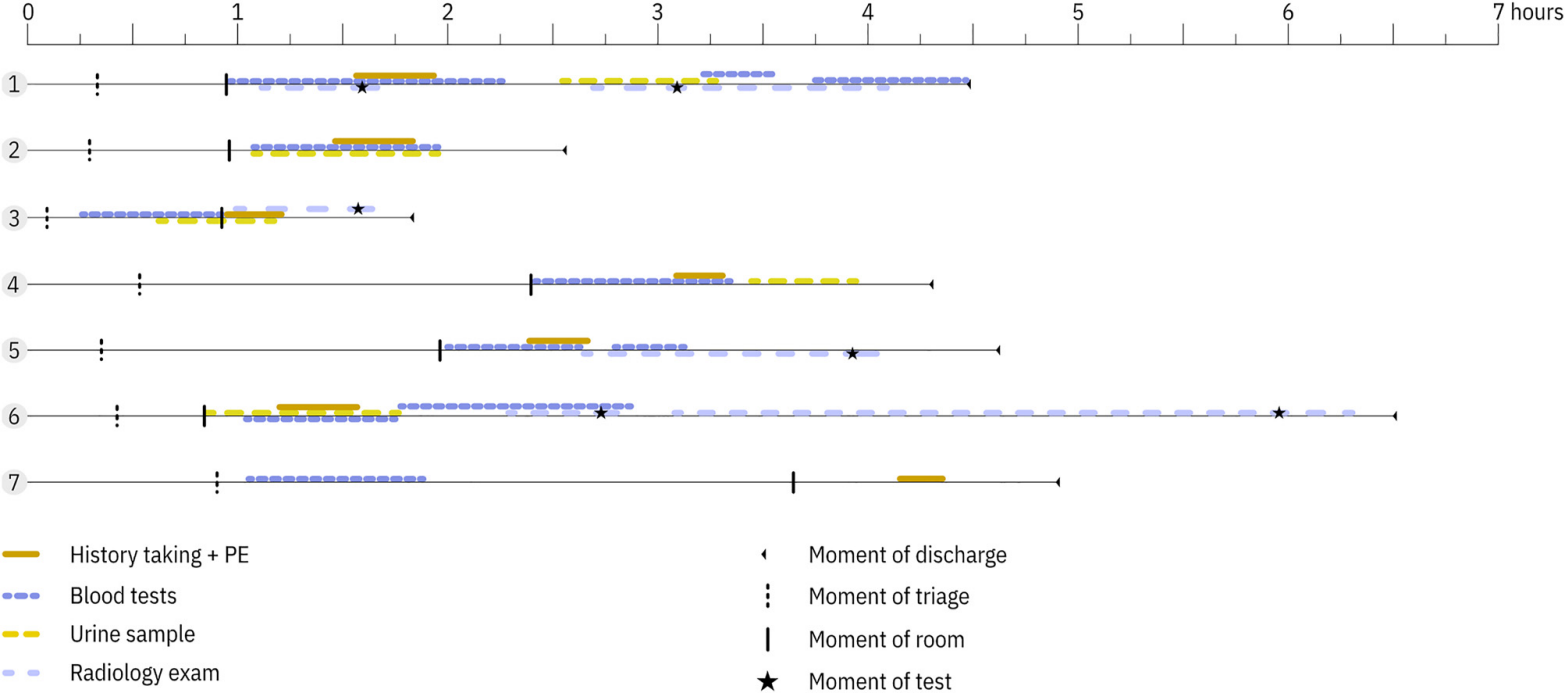
Work-as-done showing practice variation in the timing of taking of blood and urine samples in the seven patients going through triage. In cases 3 and 7, the blood samples were taken in triage. PE, physical examination.

Furthermore, variation was observed in the timing of obtaining blood samples in patients going through triage with blood samples being taken in triage in 2/7 patients (see Figure 2 and Table 2). During the co-creation session, stakeholders reached consensus that obtaining blood samples in triage could improve the ED workflow. The involved ED personnel was informed about the blood sampling during triage.

Practice variation was observed in the number of interactions within the diagnostic team: varying from 3 to 22 interactions by residents with other professionals. Discussing this practice variation, it was unclear for stakeholders whether variation in the communication within the diagnostic team was desired or undesired variation as it could be there is more communication in complex cases and variation is therefore desired. Therefore, in-depth observation of communication was postponed to a subsequent project.

### Observation cycle 3

The observations showed that the Internal Medicine consultants documented information about the preannouncement in the EHR in 75 % of the patients referred during office hours, all supervision during office hours was carried out face-to-face (100 %), and blood samples were taken in triage in 67 % (6/9) of the patients going through triage. In the co-creation session, the ED nurses and residents agreed that adding the intended diagnostics to the ‘Successful Seven’ was beneficial to the diagnostic process. Stakeholders reached consensus that face-to-face supervision during office hours and blood sampling in triage were feasible.

Practice variation was observed in the timing of serum lipase testing (see Table 2). During the co-creation session, the stakeholders agreed that adding lipase to the standard ED laboratory package for patients presenting with undifferentiated complaints could save time in the diagnostic process. Serum lipase testing was therefore imbedded within the standard laboratory package at the ED for patients presenting with undifferentiated symptoms.

Furthermore, practice variation was observed in the use of referral letters from the GP or ambulance. Discussing this practice variation, the stakeholders reached consensus that the use of referral letters was a best practice, but that implementing a practice change was unnecessary as they were already used in the majority of cases (11/15).

Besides that, speak-up behaviour from a resident towards a consultant was observed. Regarding this, the stakeholders reached consensus that it was promising behaviour, but that implementing a practice change would require action behind the scope of this project.

## Discussion

### Statement of principle findings

This study showed how a Safety-II approach can be applied to identify potential improvements in the diagnostic process. This was achieved by observing and reflecting on practice variation, followed by designing and implementing practice changes in the diagnostic process based on identified best practices. The observed practice variations and identified best practices showed that combining action research with direct observations is a suitable method to apply Safety-II in the diagnostic process in a complex and adaptive ED environment, and can lead to well-received practice changes.

In this study, we were able to learn from all 40 observed diagnostic processes instead of only learning from the five patients with misdiagnosis. The observations of the diagnostic process led to identification of eight practice variations, which were extensively discussed with stakeholders in co-creation sessions, and led to the identification of best practices. After reaching consensus on the potential impact and feasibility of these best practices, practice changes were co-designed with the stakeholder group for implementation in daily practice. This led to the implementation of five practice changes in the diagnostic process at the ED. Evaluation of the practice changes during subsequent co-creation session showed that their uptake varied from 67 to 100 %, and overall good stakeholder satisfaction.

### Strengths and limitations

A particular strength of this study is that the practice changes were made without the need to identify diagnostic errors (which has shown to be challenging) [18]. Yet, registering practice variation has shown to be an excellent basis for improving the diagnostic process. Furthermore, our action research design with direct observations can potentially be used in other clinical settings. Another strength is the involvement of patients and medical professionals directly involved in the diagnostic process during co-creation sessions. This led to the identification of best practices and development of practice changes in the diagnostic process, which were well-received as they were tailored to the everyday ED work based on the expertise of the stakeholders.

A limitation of this study is that the implemented practice changes are relevant for the ED of the participating hospital, and may not be generalisable to other hospitals. However, the use of the Safety-II approach, in an action research design, is generalisable and could elicit practice variations elsewhere. Another limitation, inherent to the qualitative study design, is that a certain level of subjectivity of the study supervisors and the stakeholder group plays a role in the identification of practice variation, and development of practice changes. Lastly, our study was not designed to formally evaluate on a large scale whether the practice changes improved the diagnostic process. However, informal small-scale evaluations of the practice changes were performed during co-creation sessions based on the observations and the experiences of the stakeholders with the practice changes, and changes were made based on their practical use. Longer term and more comprehensive analysis of the impact of our approach and the implemented practice changes are needed for formal evaluation.

### Interpretation within the context of the wider literature

To our knowledge, no other studies have used a Safety-II approach or similar approach to the diagnostic process. Based on our study findings, the Safety-II approach can unravel the complexity and variability in the diagnostic process, what is consistent with the expectations of the use of this approach in current Safety-II literature [15], 19], 21], 30]. This study adds to a scarce amount of studies bringing Safety-II concepts into medical practice, and of Safety-II studies leading to interventions improving quality of care [19], 20]. This study could catalyse additional work on using the Safety-II approach to study the diagnostic process.

Furthermore, this Safety-II study combines repetitive cycles of action research with direct observations. Most current Safety-II studies used FRAM to apply the Safety-II approach to improve processes in healthcare [31], [32], [33]. However, these studies mainly focussed on small demarcated complex processes (e.g. preoperative anticoagulation management), and the use of FRAM seems less suitable for complex and less demarcated processes (e.g. diagnostic process). Our action research design, with active stakeholder involvement in constructive co-creation sessions, led to better overall consensus on the development and implementation of practice changes, and facilitated their adoption in daily practice. Active involvement of stakeholders has shown similar results in checklist studies [34], 35].

In this study, we identified practice variation in the diagnostic process using the Safety-II approach, which resulted in the introduction of actionable practice changes in the diagnostic workflow protocols. Thereby, the implemented practice changes resulted in reducing the previously observed practice variability, what is more aligned with Safety-I thinking. However, through our iterative observations and co-creation sessions, we found that some variations reflected desirable adaptability to specific clinical circumstances and therefore were not considered suitable for standardisation. In other cases, practices initially considered promising for standardisation were later found to be context-dependent and ultimately retained as acceptable or even beneficial variations. This process illustrates that we did not seek to eliminate all variability, but rather to distinguish between variations that enhance adaptability and those that undermine safety and efficiency. Importantly, many of the identified best practices were derived from examples of care that went particularly well, which might not have been captured through a Safety-I lens alone. In recent literature, Safety-I and Safety-II are seen as distinct yet complementary to each other, and integrating them makes it possible to improve patient safety by learning from both things that go right and things that go wrong [30], 36], 37]. This is also supported by our study.

During the course of our study, we experienced a culture shift among the stakeholders to focus more on things that went well and identification of best practices in daily practice. This is in line with recent studies that concluded that looking at what goes well in everyday work (Safety-II approach) is less threatening for medical professionals compared to analysis of incidents (Safety-I approach), and will thereby contribute to a positive culture [21], 38], 39]. The ‘Successful Seven’ template for the preannouncement documentation in the EHR was adopted by seven other specialties at the ED that used similar templates adjusted to their needs. This shows how the positive approach contributed to a learning culture at the ED.

### Implications for policy, practice and research

While our study shows the potential of the Safety-II approach, also illustrated by its adoption in recent grant programs by the Agency for Healthcare Research and Quality (AHRQ), Safety-II has sparked considerable debate in safety sciences. Critics argue that Safety-II merely reframes established quality improvement practices, and focuses too heavily on the human operator, ignoring the relevance of the broader sociotechnical system [40].

## Conclusions

In conclusion, the Safety-II approach can be successfully applied to study the diagnostic process at the ED and identify potential improvements. By observing practice variations, we identified best-practices and implemented changes that were readily adopted, fostering a learning culture focused on successes rather than errors. This approach proved not only useful for immediate successes, but also sustainable with a high uptake among ED staff. Following our project, several practice changes, such as the documentation of the preannouncement, were also implemented by other medical specialties, demonstrating the potential wider applicability of our findings. Furthermore, the method shows promise for broader application in other complex clinical settings. Future research could explore the broader impact of Safety-II on diagnostic safety in other settings or on a larger scale.

## Supplementary Material

Supplementary Material
