# Supplementary material for: A Safety-II approach to learn from practice variation in the diagnostic process in the emergency department: an action research study
Source: Diagnosis (Berl). 2025 Oct 13;13(2):274–84. doi: 10.1515/dx-2025-0087 (PMC13443388; doi:10.1515/dx-2025-0087)
Supplement: Supplementary file 1 [file j_dx-2025-0087_suppl_001.docx]

**Supplementary material**

**Supplemental appendix 1**: The final version of the standardised observation form.

**- - - - - - - - - - - - - - - - - - - - - - - - - - - - - - - - - - - - - - - - - - - - - - - - - - - - - - - - - - - - - - - - - - - - - - -**

**Emergency department (ED) registration**

Time of arrival in the hospital
___________________________________________________________________________

Mode of transport to the hospital

- Ambulance
- Own transport
- Trauma helicopter

Time of receiving an ED-room
___________________________________________________________________________

The physician referring the patient to the ED

- Colleague from outpatient clinic
- Elderly care physician
- Emergency number
- General practitioner
- Other hospital
- Self-referral

Type of ED presentation

- Emergency presentation
- Representation
- Transfer from another hospital

Specialty of treating resident

- Emergency Medicine
- Internal Medicine (incl. Gastro-enterology, Hematology and Rheumatology)

Presence of medical student in diagnostic process

- No
- Yes

**- - - - - - - - - - - - - - - - - - - - - - - - - - - - - - - - - - - - - - - - - - - - - - - - - - - - - - - - - - - - - - - - - - - - - - -**

**ED preannouncement**

Was a preannouncement made to the treating resident?

- No
- Yes

Specialty of the consultant/physician making the preannouncement to the resident

- Elderly care physician (evening, night and weekend shifts)
- Emergency Medicine
- Gastro-enterology
- General practitioner (evening, night and weekend shifts)
- Hematology
- Internal Medicine
- Rheumatology

Time of receiving the preannouncement
___________________________________________________________________________

Method of communication of the preannouncement

- Documentation in electronic health record
- Face-to-face
- Phone call

Information mentioned in the preannouncement
_______________________________________________________________________________________________________________________________________________________________________________________________________________________________________________________________________________________________________________________________________________________________________________________

Other circumstances or factors influencing the preannouncement
_______________________________________________________________________________________________________________________________________________________________________________________________________________________________________________________________________________________________________________________________________________________________________________________

**- - - - - - - - - - - - - - - - - - - - - - - - - - - - - - - - - - - - - - - - - - - - - - - - - - - - - - - - - - - - - - - - - - - - - - -**

**Triage**

Time of start of triage
___________________________________________________________________________

Colour assigned during triage

- Red (immediate assessment)
- Orange (assessment <15 minutes)
- Yellow (assessment <1 hour)
- Green (assessment <3 hours)
- Blue (assessment <6 hours)

Time the patient was first seen by the treating resident after triage
___________________________________________________________________________

Other circumstances or factors influencing triage
_______________________________________________________________________________________________________________________________________________________________________________________________________________________________________________________________________________________________________________________________________________________________________________________

**- - - - - - - - - - - - - - - - - - - - - - - - - - - - - - - - - - - - - - - - - - - - - - - - - - - - - - - - - - - - - - - - - - - - - - -**

**History taking**

Time of start of history taking
___________________________________________________________________________

Function of person taking the initial history

- Consultant
- Resident
- Student

Relevant findings during history taking
___________________________________________________________________________
___________________________________________________________________________
___________________________________________________________________________
___________________________________________________________________________
___________________________________________________________________________
___________________________________________________________________________
___________________________________________________________________________
___________________________________________________________________________
___________________________________________________________________________
___________________________________________________________________________
___________________________________________________________________________
___________________________________________________________________________
___________________________________________________________________________
___________________________________________________________________________
_______________________________________________________________________________________________________________________________________________________________________________________________________________________________________________________________________________________________________________________________________________________________________________________

Duration of the history taking
___________________________________________________________________________

History taking combined with physical examination

- No
- Yes

Other circumstances or factors influencing the history taking
_______________________________________________________________________________________________________________________________________________________________________________________________________________________________________________________________________________________________________________________________________________________________________________________

**- - - - - - - - - - - - - - - - - - - - - - - - - - - - - - - - - - - - - - - - - - - - - - - - - - - - - - - - - - - - - - - - - - - - - - -**

**Physical examination**

Time of start of physical examination
___________________________________________________________________________

Physical examination by same person as history taking

- No
- Yes

Relevant findings during physical examination
____________________________________________________________________________________________________________________________________________________________________________________________________________________________________________________________________________________________________________________________________________________________________________________________________________________________________________________________________________________________________________________________________________________________________________________________________________________________________________________________________________________________________________________________________________________________________________________________________________________________________________________________________________________________________________________________________

Duration of the physical examination
___________________________________________________________________________

Duration of the history taking and physical examination
___________________________________________________________________________

Other circumstances or factors influencing the physical examination
_______________________________________________________________________________________________________________________________________________________________________________________________________________________________________________________________________________________________________________________________________________________________________________________

**- - - - - - - - - - - - - - - - - - - - - - - - - - - - - - - - - - - - - - - - - - - - - - - - - - - - - - - - - - - - - - - - - - - - - - -**

**Additional diagnostics**

Performance of blood tests

- No
- Yes

Blood tests
*Time request	Time sample taken	Time results	Relevant results*
__________	________________	________________	____________________________
____________________________

__________	________________	________________	____________________________
____________________________

__________	________________	________________	____________________________
____________________________

__________	________________	________________	____________________________
____________________________

Other circumstances or factors influencing blood tests
_______________________________________________________________________________________________________________________________________________________________________________________________________________________________________________________________________________________________________________________________________________________________________________________

**- - - - - - - - - - - - - - - - - - - - - - - - - - - - - - - - - - -**

Performance of urine tests

- No
- Yes

Urine tests
*Time request	Time sample taken	Time results	Relevant results*
__________	________________	________________	____________________________
____________________________

__________	________________	________________	____________________________
____________________________

Other circumstances or factors influencing urine tests
_______________________________________________________________________________________________________________________________________________________________________________________________________________________________________________________________________________________________________________________________________________________________________________________

**- - - - - - - - - - - - - - - - - - - - - - - - - - - - - - - - - - -**

Performance of radiology exams

- No
- Yes

Radiology exams
*Time request	Type of exam	Question	Time test	Time results	Relevant results*
__________	__________	__________	__________	__________	________________
 __________	________________
 __________	________________
__________	__________	__________	__________	__________	________________
 __________	________________
 __________	________________
__________	__________	__________	__________	__________	________________
 __________	________________
 __________	________________

Other circumstances or factors influencing radiology exams
_______________________________________________________________________________________________________________________________________________________________________________________________________________________________________________________________________________________________________________________________________________________________________________________

**- - - - - - - - - - - - - - - - - - - - - - - - - - - - - - - - - - -**

Performance of microbiology exams

- No
- Yes

Microbiology exams
*Time request	Type of exam	Time test	Time results	Relevant results*
__________	__________	__________	__________	____________________________
 ____________________________
__________	__________	__________	__________	____________________________
 ____________________________

Other circumstances or factors influencing microbiology exams
_______________________________________________________________________________________________________________________________________________________________________________________________________________________________________________________________________________________________________________________________________________________________________________________

**- - - - - - - - - - - - - - - - - - - - - - - - - - - - - - - - - - -**

Performance of an electrocardiogram (ECG)

- No
- Yes

Electrocardiogram
*Time request	Time of ECG	Time of assessment	Relevant results*
__________	__________	________________	__________________________________
 __________________________________
__________	__________	________________	__________________________________
 __________________________________

Other circumstances or factors influencing electrocardiograms
_______________________________________________________________________________________________________________________________________________________________________________________________________________________________________________________________________________________________________________________________________________________________________________________

**- - - - - - - - - - - - - - - - - - - - - - - - - - - - - - - - - - - - - - - - - - - - - - - - - - - - - - - - - - - - - - - - - - - - - - -**

**Communication**

Communication moments (excl. history taking)

*Time	Consultation by/of	Method (e.g. call)	Discussed information (supervision *)*
____	________________	_______________	__________________________________
__________________________________
__________________________________
__________________________________
 __________________________________
____	________________	_______________	__________________________________
__________________________________
__________________________________
__________________________________
 __________________________________
____	________________	_______________	__________________________________
__________________________________
__________________________________
__________________________________
 __________________________________
____	________________	_______________	__________________________________
__________________________________
__________________________________
__________________________________
 __________________________________
____	________________	_______________	__________________________________
__________________________________
__________________________________
__________________________________
 __________________________________
____	________________	_______________	__________________________________
__________________________________
__________________________________
__________________________________
 __________________________________
____	________________	_______________	__________________________________
__________________________________
__________________________________
__________________________________
 __________________________________
____	________________	_______________	__________________________________
__________________________________
__________________________________
__________________________________
 __________________________________
____	________________	_______________	__________________________________
__________________________________
__________________________________
__________________________________
 __________________________________
____	________________	_______________	__________________________________
__________________________________
__________________________________
__________________________________
 __________________________________
____	________________	_______________	__________________________________
__________________________________
__________________________________
__________________________________
 __________________________________
____	________________	_______________	__________________________________
__________________________________
__________________________________
__________________________________
 __________________________________
____	________________	_______________	__________________________________
__________________________________
__________________________________
__________________________________
 __________________________________
____	________________	_______________	__________________________________
__________________________________
__________________________________
__________________________________
 __________________________________
____	________________	_______________	__________________________________
__________________________________
__________________________________
__________________________________
 __________________________________
____	________________	_______________	__________________________________
__________________________________
__________________________________
__________________________________
 __________________________________
____	________________	_______________	__________________________________
__________________________________
__________________________________
__________________________________
 __________________________________
____	________________	_______________	__________________________________
__________________________________
__________________________________
__________________________________
 __________________________________

Other circumstances or factors influencing the communication in the diagnostic process
_______________________________________________________________________________________________________________________________________________________________________________________________________________________________________________________________________________________________________________________________________________________________________________________

- - - - - - - - - - - - - - - - - - - - - - - - - - - - - - - - - - - - - - - - - - - - - - - - - - - - - - - - - - - - - - - - - - - - - - -

**Differential diagnosis**

Time of formulation of first differential diagnosis
___________________________________________________________________________

First differential diagnosis
_______________________________________________________________________________________________________________________________________________________________________________________________________________________________________________________________________________________________________________________________________________________________________________________

Changes made in differential diagnosis

- No
- Yes

Changes in differential diagnosis
*Time	Reason for change	New differential diagnosis*
__________	________________	_____________________________________________
 _____________________________________________
 _____________________________________________
 _____________________________________________
 _____________________________________________
__________	________________	_____________________________________________
 _____________________________________________
 _____________________________________________
 _____________________________________________
 _____________________________________________
__________	________________	_____________________________________________
 _____________________________________________
 _____________________________________________
 _____________________________________________
 _____________________________________________

Other circumstances or factors influencing the differential diagnosis
_______________________________________________________________________________________________________________________________________________________________________________________________________________________________________________________________________________________________________________________________________________________________________________________

- - - - - - - - - - - - - - - - - - - - - - - - - - - - - - - - - - - - - - - - - - - - - - - - - - - - - - - - - - - - - - - - - - - - - - -

**Working diagnosis**

First working diagnosis *Time	Working diagnosis*
__________	_______________________________________________________________
 _______________________________________________________________

Changes made in working diagnosis

- No
- Yes

Changes in working diagnosis  *Time	Reason for change	New working diagnosis*
__________	________________	_____________________________________________
 _____________________________________________
 _____________________________________________
 _____________________________________________
 _____________________________________________
__________	________________	_____________________________________________
 _____________________________________________
 _____________________________________________
 _____________________________________________
 _____________________________________________

Other circumstances or factors influencing the working diagnosis
_______________________________________________________________________________________________________________________________________________________________________________________________________________________________________________________________________________________________________________________________________________________________________________________

- - - - - - - - - - - - - - - - - - - - - - - - - - - - - - - - - - - - - - - - - - - - - - - - - - - - - - - - - - - - - - - - - - - - - - -

**Treatment plan**

Final treatment plan
*Time	Treatment plan*
__________	_______________________________________________________________
 _______________________________________________________________
 _______________________________________________________________
 _______________________________________________________________
 _______________________________________________________________

End time of the diagnostic process
__________________________________________________________________________

Other circumstances or factors influencing the treatment plan
_______________________________________________________________________________________________________________________________________________________________________________________________________________________________________________________________________________________________________________________________________________________________________________________

- - - - - - - - - - - - - - - - - - - - - - - - - - - - - - - - - - - - - - - - - - - - - - - - - - - - - - - - - - - - - - - - - - - - - - -

**Use of information resources**

Use of information resources in diagnostic process

- No
- Yes

Used information resources *Time	Name resource	Used information*
__________	________________	_____________________________________________
 _____________________________________________
 _____________________________________________
 _____________________________________________
 _____________________________________________
__________	________________	_____________________________________________
 _____________________________________________
 _____________________________________________
 _____________________________________________
 _____________________________________________
__________	________________	_____________________________________________
 _____________________________________________
 _____________________________________________
 _____________________________________________
 _____________________________________________

Other circumstances or factors influencing the use of information resources
_______________________________________________________________________________________________________________________________________________________________________________________________________________________________________________________________________________________________________________________________________________________________________________________

- - - - - - - - - - - - - - - - - - - - - - - - - - - - - - - - - - - - - - - - - - - - - - - - - - - - - - - - - - - - - - - - - - - - - - -

**Other**

Additional factors or circumstances potentially influencing the diagnostic process
__________________________________________________________________________________________________________________________________________________________________________________________________________________________________________________________________________________________________________________________________________________________________________________________________________________________________________________________________
___________________________________________________________________________
_________________________________________________________________________________________________________________________________________________________________________________________________________________________________

**Supplemental appendix 2**: Explanation of the different items in the ‘Successful Seven’ template for the documentation of the preannouncement in the electronic health record.

| Item of Successful Seven | Explanation |
| --- | --- |
| Referring physician | The function of the physician who referred the patient to the ED (e.g. colleague from outpatient clinic, elderly care physician or general practitioner). |
| Reason for ED presentation | The main reason for ED presentation (e.g. rule out pulmonary embolisms, analysis for hypoxemia with unknown cause, or hyponatremia). |
| Presenting complaints | In short, the main complaints of the patient as described by the referring physician (e.g. shortness of breath since three days, slowly progressive over time). |
| Relevant medical history/medication | Any medical history/medication relevant for the ED presentation or related to the presenting complaints (e.g. the use of immunosuppressive medication). |
| Differential diagnosis | The differential diagnosis according to the consultant taking the preannouncement phone call from the referring physician (e.g. pneumonia or pulmonary embolisms). |
| Intended diagnostic tests and follow-up | The required diagnostic tests (blood tests, urine tests, radiology exams) and the expected ED disposition (e.g. discharge home or hospitalisation). |
| Code status | The resuscitation code status, describing the type of resuscitation procedures (cardiopulmonary resuscitation, mechanical ventilation, and ICU-admission). |

*ED: Emergency department; ICU: Intensive care unit*
